# High-Fat Diet Increases HMGB1 Expression and Promotes Lung Inflammation in Mice Subjected to Mechanical Ventilation

**DOI:** 10.1155/2018/7457054

**Published:** 2018-02-12

**Authors:** Ana Beatriz Farias de Souza, Máira Tereza Talma Chírico, Christiane Teixeira Cartelle, Guilherme de Paula Costa, André Talvani, Sílvia Dantas Cangussú, Rodrigo Cunha Alvim de Menezes, Frank Silva Bezerra

**Affiliations:** ^1^Department of Biological Sciences (DECBI), Center of Research in Biological Sciences (NUPEB), Federal University of Ouro Preto (UFOP), Ouro Preto, MG, Brazil; ^2^Department of Pathology, Institute of Biological Sciences, Federal University of Minas Gerais, Belo Horizonte, MG, Brazil

## Abstract

This study aims to evaluate the effects of a high-fat diet and mechanical ventilation on the pulmonary and systemic inflammatory response in C57BL/6 mice. Male C57BL/6 mice were divided into two groups: one received a standard diet, and the other received a high-fat diet. After 10 weeks, the groups were further divided into two groups each: control group (CG), mechanical ventilation group (MVG), diet group (DG), and diet mechanical ventilation group (DMVG). MVG and DMVG underwent mechanical ventilation for 60 minutes. All animals were euthanized for subsequent analysis. Animals receiving a high-fat diet presented higher body mass, adipose index, and greater adipocyte area. In the lung, the expression of HMGB1 was greater in DG and DMVG than in CG and MVG. CCL2 and IL-22 levels in MVG and DMVG were increased compared to those in CG and DG, whereas IL-10 and IL-17 were decreased. Superoxide dismutase activity was higher in MVG and DMVG than in CG. Catalase activity was lower in DG than in CG, and in MV groups, it was lower than that in CG and DG. MV and obesity promote inflammation and pulmonary oxidative stress in adult C57BL/6 mice.

## 1. Introduction

The prevalence of obesity is increasing globally at alarming rates, and according to the World Health Organization, the worldwide prevalence of obesity has more than doubled since 1980 and is associated with several comorbidities [1, 2]. Although its pathogenesis is not completely understood, the development of obesity does not involve a single cause but a complex combination of several conditions caused by multiple factors resulting in the disease phenotype. The etiology of obesity involves genetic factors, which can be attenuated or exacerbated by dietary, environmental, and psychosocial factors [3]. Obesity, particularly the expansion of visceral adipose tissue, promotes increased production of adhesion molecules, recruitment and differentiation of monocytes, and, consequently, elevated production of cytokines and adipokines [1, 4]. Moreover, adipose tissue accumulation in the thoracic cavity and abdomen alters respiratory physiology causing an increased load on the respiratory musculature, leading to decreased chest wall compliance and increased resistance, altered ventilation perfusion relation, lung volume, and capacity [5–7]. Recent data indicate that in intensive care units, approximately 20% of patients are obese or severely obese [8]. These patients have more complications and need longer hospitalization and mechanical ventilation [5].

Mechanical ventilation (MV) is a tool used for patients with respiratory insufficiency. Although it presents an important therapeutic role, MV can cause lung injury or aggravate preexisting injury, resulting in ventilator-induced lung injury (VILI) [9]. The mechanisms by which VILI develops are not fully described, but studies demonstrate that cyclic stretching caused by MV may damage alveolar epithelial cells and increase permeability, which is associated with leukocyte recruitment into the air space, resulting in the production of inflammatory cytokines and reactive oxygen species (ROS) [10]. ROS production at high concentrations is related to redox imbalance, characterized by an altered ratio of oxidants to antioxidants, where the concentration of reactive species transiently or chronically increases the damage in the regulation of cellular metabolism, thus injuring the cellular constituents [11, 12]. Despite the clinical and epidemiological relevance, few studies have explored the association between obesity and MV and its deleterious effects on the body. In this study, we evaluated the effects of a high-fat diet and mechanical ventilation on redox imbalance and on the pulmonary and systemic inflammatory response in C57BL/6 mice.

## 2. Methods

### 2.1. Animals

Forty male C57BL/6 mice, aged 7 to 8 weeks, were obtained from the Animal Science Center (CCA) of the Federal University of Ouro Preto (UFOP). The animals were kept under controlled conditions of temperature (21 ± 2°C), humidity (50 ± 10%), and luminosity (12 hours light/dark cycle). The experimental procedures were performed in accordance with the Ethical Principles of Animal Experimentation established by the Ethics Committee on Animal Use (CEUA) of UFOP and approved by protocol number 2015/41.

### 2.2. Experimental Design and Diet Composition

First, the animals were divided into two groups (*n* = 20) according to the type of diet provided: control group and diet group. The control group received standard chow (Nuvilab®) containing 3.5% lipids; the diet group was provided a high-fat diet with 45% lipids (based on formula D12451, Research Diets Inc., New Brunswick, NJ, USA) [13]. The animals were evaluated weekly for weight gain on a digital balance (Marte Scientific and Industrial Instrumentation®, São Paulo, BR). At the end of 10 weeks of this period, the animals from each group were randomly divided into two groups of 10 animals each: control group (CG), mechanical ventilation group (MVG), diet group (DG), and diet mechanical ventilation group (DMVG). Animals from the CG and DG were maintained on spontaneous ventilation, and MVG and DMVG were subjected to mechanical ventilation for 60 minutes.

### 2.3. Mechanical Ventilation

Twenty-four hours after the end of the nutritional protocol, the animals of the MVG and DMVG were sedated and anesthetized by intraperitoneal administration of ketamine (10 mg/kg) and xylazine (8 mg/kg) and placed on a surgical table for a median incision to be performed in the anterior cervical region. The musculature was dissected with the aid of a hemostatic forceps, the trachea was exposed, and an incision was made with a catheter to connect the animal to the MiniVent ventilator (Harvard Apparatus, Massachusetts, USA). The animals were ventilated in the volume-controlled mode according to the following parameters: tidal volume of 7 mL/kg, respiratory rate of 150 breaths/minute, and inspired fraction of oxygen at 21%. All animals subjected to mechanical ventilation were paralyzed using suxamethonium chloride (0.3 mg/kg, intravenous). Peripheral saturation and control of body temperature were assessed throughout the period for which the animals were ventilated.

### 2.4. Euthanasia

After the mechanical ventilation in the experimental protocol, blood was collected by cardiac puncture and placed in polypropylene tubes containing 15 *μ*L of anticoagulant for the evaluation of hematological parameters using an electronic counting device (Mindray® Bio-Medical Electronics Co. Ltd., Shenzhen, China) [14].

### 2.5. Collection and Analysis of Bronchoalveolar Lavage Fluid (BALF)

Immediately after euthanasia, the thorax of each animal was opened, the left main bronchus was clamped, the trachea was cannulated, and the left lung was washed with 1.5 mL of saline solution (3 × 500 *μ*L). The samples were kept on ice until the end of the procedure to avoid cell lysis. A Neubauer chamber was used for the total leukocyte count of BALF. To determine the differential cell count, 250 *μ*L samples were centrifuged in a cytocentrifuge (INBRAS health equipment, São Paulo, BR) and stained with a fast panoptic coloration kit (Laborclin, Paraná, BR), and 100 cells per slide were counted; each procedure was performed by two evaluators [15].

### 2.6. Tissue Processing and Homogenization

After BALF collection, the right ventricle was perfused with saline solution to remove blood from the lungs. The right lung was clamped, and the left lung instilled with 4% buffered formalin (pH 7.2) at a pressure of 25 cmH_2_O for 2 minutes, via the trachea. The left lung was then removed and immersed in fixative solution for 48 hours. The samples were processed, and slides were stained with hematoxylin and eosin (H&E) to perform stereological analyses or used for immunohistochemistry. The right lung was homogenized with 1.5 mL of phosphate buffer (pH 7.4); the samples were centrifuged for 10 minutes at 10,000 rpm; the supernatant was collected and stored at −80°C for biochemical analysis [14].

### 2.7. Calculation of Body Fat Index

The adipose tissue (mesenteric, retroperitoneal, and epididymal) was removed and weighed to determine the body fat index, which was calculated according to the equation described by Catta-Preta et al. [16].

### 2.8. Morphometric Analyses of Lung and Adipose Tissue

Morphometric analyzes were performed on lungs and epididymal adipose tissue stained with H&E. Twenty random fields of lung histological slides were photographed using a light microscope equipped with a Leica BM5000 digital camera (Leica DFC 300 FX) coupled to the Leica Application Suite image capture software using a 40x microscopic objective. The volume density analysis of the alveolar septum (Vv) was performed in a test system composed of 16 points and a known test area, as described by Mandarim-de-Lacerda [17] and Campos et al. [15].

For analyzing the adipocyte area, the histological sections were photographed using the same microscope with a 10x microscopic objective. Ten random fields were analyzed, and the mean area of the adipocytes was obtained by analyzing 50 adipocytes per slide in ImageJ 1.6.0 software (Wayne Rasband—National Institutes of Health, USA) [14, 18].

### 2.9. Immunohistochemistry

Two histological sections of each animal were stained with HMGB1 (EPR3507) (Abcam, UK) by immunohistochemistry. The slides were deparaffinized in xylene and rehydrated in decreasing ethanol concentrations. Subsequently, antigen recovery was performed with EDTA solution pH 9.0 in a water bath at 96°C for 20 minutes. Endogenous peroxidase activity was blocked with H_2_O_2_ solution in methanol, twice for 15 minutes. Nonspecific binding was blocked with 3% skim milk (MOLICO, Nestlé Brasil Ltda., Araçatuba, SP) in phosphate buffered saline (PBS), followed by blocking with 2% BSA (bovine serum albumin; Inlab, Brazil) in PBS and blocking with normal goat serum (NGS) diluted in PBS at 1 : 20. The slides were incubated for 30 minutes in each blocking solution and washed in PBS after each incubation period, except for the blocking performed with NGS. The slides were incubated overnight at 4°C with rabbit primary monoclonal anti-HMGB1 diluted at 1 : 250. After incubation with the primary antibody, the slides were washed three times with PBS, followed by incubation with the biotinylated secondary antibody in a humid chamber at 37°C for 30 minutes. Finally, the slides were incubated under the same conditions with the streptavidin/peroxidase complex (Dako, Santa Clara, CA, USA). Peroxidase activity was detected using the 3,3-diaminobenzidine substrate (DAB). All slides were stained with Harris hematoxylin. For each histological section stained with HMGB1, a control was prepared in which the primary antibody was suppressed.

Morphometric analysis of the sections stained by immunohistochemistry was performed in 20 random fields of the slides photographed at a magnification of 20x using ImageJ 1.6.0 software (Wayne Rasband—National Institutes of Health, USA). In each field, the total number of nuclei and the number of nuclei labeled for the antibody used were counted, and the ratio of labeled nuclei/total nuclei was calculated [19].

### 2.10. Analysis of Antioxidant Defense and Biomarkers of Oxidative Stress

Superoxide dismutase activity was measured in the tissue homogenate according to the method described by Marklund and Marklund [20], which is based on the ability of SOD to inhibit pyrogallol autoxidation. Catalase activity was measured according to the method described by Aebi [21] from the decreased H_2_O_2_ at an absorbance of 240 nm. Glutathione dosage was adapted from a Sigma commercial kit (CS0260; Sigma, St. Louis, MO, USA), which uses a kinetic method to measure the total glutathione levels (GSH + GSSG) in biological samples by reducing 5,5′-dithio-bis-(2-nitrobenzoic acid) to 5-thio-2-nitrobenzoic acid [22]. Formation of thiobarbituric acid reactive substances (TBARS) was used to measure lipid peroxidation; the method is based on the ability of thiobarbituric acid to bind to oxidized lipids as previously described by Buege and Aust [23]. For determination of carbonylated proteins, a protocol adapted from the method described by Reznick and Packer [24] was used. The total protein content in the samples was determined by the Bradford method [25].

### 2.11. Immunoenzymatic Assay for Inflammatory Markers

The pulmonary homogenate was used for analyses of monocyte chemoattractant protein-1 (MCP-1 or CCL2), regulated on activation, normal T cell expressed and secreted (RANTES or CCL5), and interleukins 10, 17, and 22. The assays were performed in 96-well plates; 100 *μ*L of monoclonal antibody was added to the protein (or peptide), which was diluted in PBS containing 0.1% bovine serum albumin (BSA; Sigma-Aldrich, Billerica, MA, USA). After incubation for 12 hours at 37°C, the plates were blocked with 300 *μ*L/well of a PBS solution containing 1% BSA for 1 hour at 37°C. The samples were applied in a volume of 100 *μ*L to each well. The staining intensity was measured using an enzyme-linked immunosorbent assay (ELISA) reader at a wavelength of 490 nm. All ELISA kits were purchased from PeproTech (Ribeirão Preto, Brazil).

### 2.12. Statistical Analysis

Parametric distribution of the data was evaluated using the Kolmogorov-Smirnov normality test. Parametric data were expressed as mean and standard error of the mean; nonparametric data were expressed as median, minimum, and maximum values. Analysis of the body mass data was performed by two-way ANOVA by the Bonferroni posttest. For comparison of two or more groups, a one-way ANOVA followed by Tukey's posttest was used for comparing two or more groups. For nonparametric data, the Kruskal-Wallis test was applied with Dunn's posttest. Significant differences were considered at *p* < 0.05. All statistical analyses were performed using GraphPad Prism software 5.0 (San Diego, CA, USA).

## 3. Results

### 3.1. Effects of High-Fat-Diet Administration

**Figure 1:**
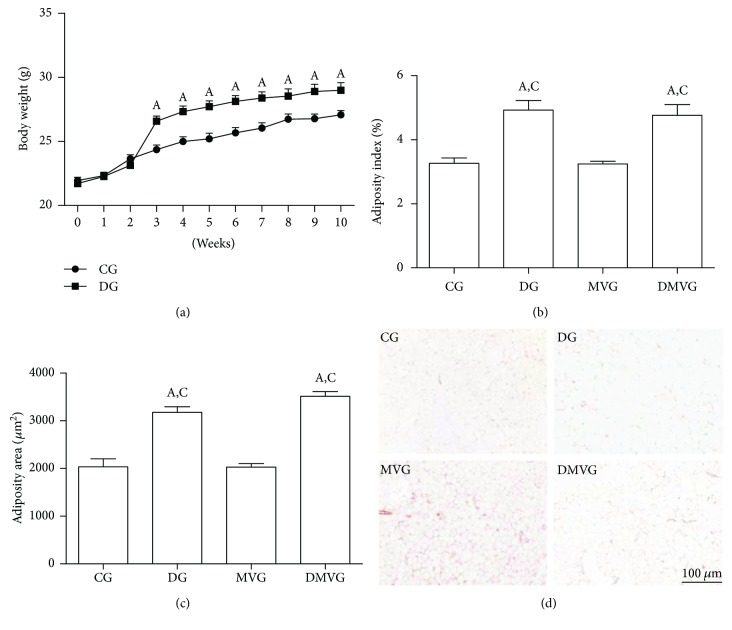
Effects of the hyperlipidic diet on body mass and adipose tissue. (a) Body mass gain over the 10-week experiment. (b) Body adiposity index. (c) Area of adipocytes. (d) Histological section of epididymal adipose tissue stained with hematoxylin and eosin. Bar = 100 *μ*m. For (a), data are expressed as mean ± standard error of the mean (*n* = 20). (a) represents difference compared to the control group *p* < 0.05 using two-way ANOVA followed by the Bonferroni posttest. For (b) and (c), (A) and (C) represent a significant difference in relation to CG and MVG. Data are expressed as mean ± standard error of the mean (*n* = 10). Analysis was performed by one-way ANOVA followed by Tukey's posttest (*p* < 0.05).

Since the third week of the experiment, the diet group presented higher body mass than did the group that received a standard diet. The difference between the groups was maintained until the end of the experiment (Figure 1(a)). The animals in DG and DMVG presented higher adiposity index and adipocyte area than did those in to CG and MVG (*p* < 0.0001) (Figures 1(b)–1(d)).

### 3.2. Total and Differential Leukocyte Count in Blood

In peripheral blood, the animals in MVG and DMVG presented a higher leukocyte count than did those in CG and DG (*p* < 0.0001, *F* = 11.5). The neutrophil count was higher in MVG and DMVG than in CG and DG; in MVG, the count was higher than that in DMVG (*p* < 0.0001, *F* = 181.2). The monocyte count was higher in groups subjected to MV than that in CG and DG (*p* < 0.0001, *F* = 15.0) (Table 1).

### 3.3. Cell Recruitment to Bronchoalveolar Lavage Fluid

High-fat diet, mechanical ventilation, and the combination of diet and ventilation caused a higher recruitment of cells to the lung when compared to that in the control group (*p* < 0.0001, *F* = 109.8). The animals in DG had a higher macrophage population in BALF than had those in CG (*p* < 0.0001, *F* = 76.8). The number of macrophages, neutrophils, and lymphocytes in MVG and DMVG was increased in comparison to that in CG and DG (*p* < 0.0001, *F* = 24.9) (Table 2).

### 3.4. Evaluation of Cytokine and Chemokine Levels in the Pulmonary Parenchyma

The inflammatory markers CCL2, CCL5, IL-17, IL-22, and IL-10 were analyzed in the lung parenchyma to assess the inflammatory state of the lungs. The levels of CCL2 (*p* < 0.0001, *F* = 13.1) and IL-22 (*p* < 0.0001, *F* = 14.6) were higher in the groups subjected to MV compared to those in the animals on spontaneous ventilation whereas the levels of IL-17 (*p* < 0.0001, *F* = 11.6) and IL-10 (*p* < 0.0001, *F* = 15.4) were lower in these groups (Table 3).

### 3.5. Effects of Mechanical Ventilation and Obesity on Oxidative Stress Biomarkers

Lipid peroxidation in MVG was higher than that in the other experimental groups (*p* = 0.0004, *F* = 7.9). The protein oxidation levels were higher in MVG and DMVG than those in CG and DG (*p* < 0.0001, *F* = 18.6). Regarding the activity of antioxidant enzymes, SOD activity was higher in MVG and DMVG than in CG (*p* = 0.0067, *F* = 5.0). CAT activity was lower in DG than in CG, and in groups subjected to MV, enzyme activity was even lower in comparison with that in CG and DG (*p* < 0.0001, *F* = 117.1). The GSH/GSSG ratio was lower in MVG than in CG (*p* < 0.03, *F* = 3.4) (Table 4).

### 3.6. Analysis of HMGB1 in Lung Parenchyma

**Figure 2:**
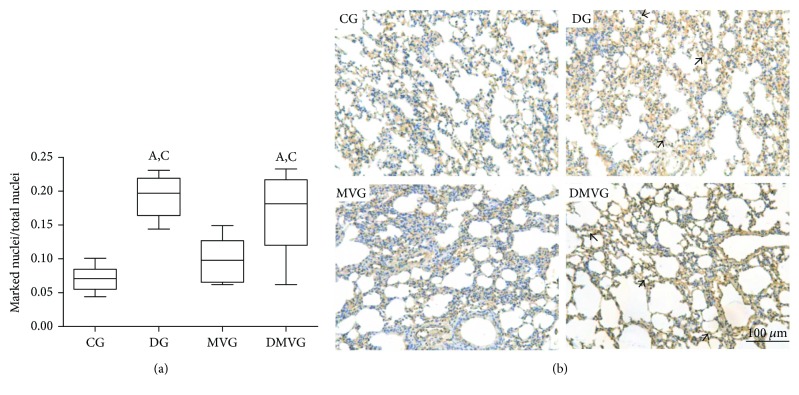
Immunohistochemistry for HMGB1. (a) Ratio between the number of nuclei labeled for HMGB1 antibody and the total number of nuclei. Data are expressed as mean ± standard error of the mean (*n* = 10). (A) and (C) represent a significant difference in relation to CG and MVG (*p* < 0.05) using Kruskal-Wallis analysis followed by Dunn's posttest. (b) Histological section of lung parenchyma stained by immunohistochemical technique. Bar = 100 *μ*m. The arrows point to marked nuclei.

The morphometric analysis of HMGB1 immunohistochemistry showed a higher number of nuclei labeled with the antibody in DG and DMVG than in CG and MVG, which can be observed by the highest ratio of marked nuclei/total nuclei (Figure 2).

### 3.7. Morphometric Evaluation of the Pulmonary Parenchyma

**Figure 3:**
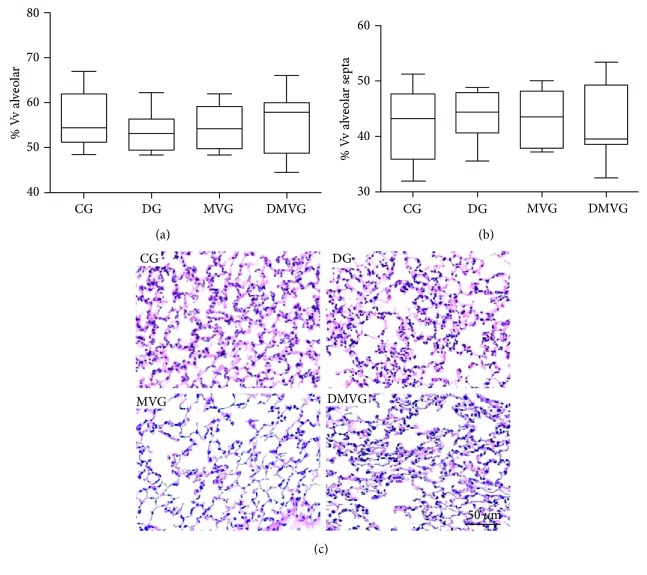
Stereological analyses of lung sections. (a) Volume density of alveolar septa. (b) Volume density of alveolar airspace. (c) Histological section of lung parenchyma stained with H&E. Bar = 50 *μ*m.

The stereological analysis showed no differences in volume density of alveolar air (Vv[a]) and in volume density of alveolar septa (Vv[sa]) (Figure 3).

## 4. Discussion

In this study, we evaluated the effects of a high-fat diet and mechanical ventilation on the inflammatory response and redox imbalance. The effects of diet were observed under body mass, adiposity index and adipocyte area, influx of cells to the lung parenchyma, hematological parameters, oxidative stress analysis, and inflammatory markers in the lungs.

Studies have shown that the composition of the diet offered in experimental models influences the development of obesity and the diseases associated with obesity, since nutrients act as cellular signals [26, 27]. In our study, animals that received the high-fat diet presented higher body mass, increased body adiposity, and greater area of adipocytes than did the control animals. This diet has been previously used in experimental models for the induction of obesity and is accompanied by an increase in body mass [16, 28]. This increase is directly related to the increase in adiposity and the greater area of adipocytes, since the excessive caloric intake is associated with storage of excess energy in adipose tissue in the form of lipids, leading to its expansion [16, 29].

Obesity is characterized by high body mass and also by systemic and local inflammatory changes, with an increase in the production of proinflammatory cytokines [1]. In this context, the role of HMGB1 protein in tissue inflammation has been studied. In our study, we observed that animals receiving a high-fat diet showed a higher number of nuclei labeled for the HMGB1 protein. There are no published studies demonstrating the expression of this protein in lungs in an experimental model of obesity, but some previous work has shown that obesity is associated with increased expression of HMGB1 and inflammatory cytokines in the adipose tissue [30, 31]. We believe that our finding is related to the chronic and systemic inflammatory state caused by obesity and may be related to the still-unknown role of HMGB1 in disease development [31].

Both obesity and mechanical ventilation have been shown to generate inflammatory processes [14, 32]. In order to evaluate whether mechanical ventilation and a diet rich in saturated lipids caused an inflammatory response, the leukocytes in blood and BALF were examined. Our results demonstrate that both insults generated an inflammatory response. Animals that received the high-fat diet presented higher recruitment of macrophages to the pulmonary parenchyma, which can be explained by the fact that these cells are fundamental regulators of immune responses and inflammation in obesity [33]; our results corroborate the findings of Tashiro et al. [34] who observed an increased number of macrophages in the bronchoalveolar lavage of mice provided a high-fat diet. In groups subjected to MV, there was an increase in the macrophages, neutrophils, and lymphocytes in BALF, and the monocytes and neutrophils in blood. Some studies have shown that macrophages are involved in the initial phase of lung injury through production of inflammatory mediators or by changes in barrier function [35, 36]. Possibly, the recruitment of macrophages to the site of inflammation altered the alveolar permeability, which resulted in recruitment of neutrophils to the lungs. The presence of neutrophils in the airspace is a consistent feature of lung injury in animals and humans because these are the first cells of the immune system to be recruited to the site of inflammation [37]. In addition, we observed that MV led to recruitment of lymphocytes to the lung; our results corroborate the findings of Chess et al. [38] who reported a higher percentage of lymphocytes in ventilated animals with a moderate tidal volume.

Cytokines and chemokines are produced at the site of inflammation by different cells of the innate and adaptive immune system, such as monocytes and neutrophils [1]. Using immunoenzymatic assays performed on lung homogenates, we found decreased IL-17 and increased IL-22 levels in the groups subjected to MV. Interleukin-17 is a proinflammatory interleukin involved in the recruitment of neutrophils to the site of inflammation [39]. Interleukin-22 may have anti- or proinflammatory functions: in the presence of IL-17, IL-22 promotes inflammation in the airways, but in the absence of this IL-17, IL-22 may have a protective role in the airways [40]. Previous studies have shown that IL-22 administration has protective effects in lung injury [41, 42]. In our mechanical ventilation model, interleukin-22 exerts a protective action, as it is increased in groups showing decreased IL-17 levels. IL-22 is related to recruitment of the innate immune cells and increased chemokine production [43]. In our study, the ventilated animals presented with increased CCL2. Ikeuchi et al. [44] observed that IL-22 induced CCL2 expression in rheumatoid arthritis, and studies have shown increased CCL2 levels in animals ventilated with moderate tidal volumes [45]. Our results suggest that CCL2 production was increased for IL-22 to repair the possible tissue damage caused by the antiphysiological mechanism of MV.

In our study, the groups subjected to MV presented a decrease in IL-10 compared to the animals kept on spontaneous ventilation. IL-10 is an anti-inflammatory cytokine that may decrease or inhibit the synthesis or secretion of inflammatory factors [46]. Some studies have observed a reduction in IL-10 and an increase in the production of inflammatory cytokines in an experimental model and clinical trial of mechanical ventilation [47, 48]. Considering previous findings, we believe that the acute inflammatory process triggered by MV leads to a reduction in interleukin production, which may contribute to the development of lung injury.

The recruitment of inflammatory cells to the pulmonary parenchyma is related to the production of oxidants and may induce acute lung injury [49]. Kavazis et al. [50] demonstrated that mechanical ventilation leads to oxidative stress. We analyzed the oxidative damage, and in the two groups submitted to MV, increased protein oxidation was observed. In addition we analyzed lipid oxidation, and there are two main ways to assess lipid peroxidation: by measuring the formation of alkoxyl radicals, as analyzed in this study, and by measuring the formation of the peroxyl radical [51]. In this study, in the animals of the mechanical ventilation group (MVG), we observed only the effect of mechanical ventilation in the lungs of the mice; however, the animals in the diet mechanical ventilation group (DMVG) previously ventilated received the high-fat diet for 10 weeks. Thus, due to the nutritional protocol to which the animals were submitted to, lipid oxidation may have occurred through the formation of isoprostanes. Chacon-Cabrera et al. [52] suggested that MV leads to oxidative damage only when used with a nonphysiological tidal volume. Our results differ from those of previous studies, as we have shown that in animals without prior lung injury, mechanical ventilation leads to oxidative stress. In order to counterbalance the reactive species, the lungs present an antioxidant defense system that includes the SOD, CAT, and GPx enzymes [53]. In our study, we observed an increase in the inflammatory cells in the pulmonary parenchyma in groups submitted to mechanical ventilation, and it is known that macrophages and neutrophils contribute to the increase in the production of reactive oxygen species. In this context, the groups subjected to MV showed an increase in SOD activity. The increase of superoxide dismutase activity can be observed, since SOD is one of the first enzymes of the antioxidant defense system to act in the removal of the reactive species [54]. Concentrations of hydrogen peroxide have increased as a result of higher SOD activity. Hydrogen peroxide removal will occur by the action of both catalase and glutathione peroxidase [54]. In our study, we observed a decrease in CAT activity and GSH/GSSG ratio. Marín-Corral et al. [55] observed that in animals ventilated with a moderate tidal volume, there was a reduction in catalase activity. Previous studies have reported a reduction in catalase activity in the lung and liver of rats fed a high-fat diet [56, 57]. The result of our study corroborates that of the previous studies; we believe that mechanical ventilation and consumption of a diet rich in saturated lipids altered oxygen metabolism, increasing the production of reactive species, which led to depletion of catalase reserves [58]. Andrade et al. [59] and Pires et al. [60] demonstrated a reduction in GSH/GSSG ratio in rats and mice submitted to mechanical ventilation, using a tidal volume similar to the used in this study. Reddy et al. [61] reported that exposure of epithelial cells to cyclic stretching caused a significant reduction in this ratio.

The changes in pulmonary histoarchitecture are mediated by protein oxidation, peroxidation of membrane lipids, and DNA strand breakage; inflammatory cells such as macrophages and neutrophils are also involved in lung architecture remodeling [62, 63]. In our study, although the results showed cell recruitment and oxidative damage, we did not find any alterations in pulmonary histoarchitecture; we believe that the short time of mechanical ventilation and the ventilation strategy used might have influenced this result.

## 5. Conclusions

The present study has some limitations; we could not analyze the ventilatory mechanics and hemodynamics of the animals on ventilation. These data would allow the determination of the influence of the high-fat diet and the mechanical ventilation on respiratory physiology. However, for our data set, it is possible to conclude that the mechanical ventilation and its association with obesity promoted inflammation and pulmonary oxidative stress in adult mice.

## Figures and Tables

**Table 1:** Total and differential evaluation of blood cells from experimental groups.

	CG	DG	MVG	DMVG
Leukocytes (×10^3^/mL)	2.61 ± 0.37	2.14 ± 0.22	4.72 ± 0.35^a,b^	3.90 ± 0.36^a,b^
Lymphocytes (×10^3^/mL)	2.36 ± 0.29	1.76 ± 0.20	2.15 ± 0.22	2.02 ± 0.20
Neutrophils (×10^3^/mL)	0.06 ± 0.01	0.06 ± 0.01	0.92 ± 0.01^a,b,d^	0.38 ± 0.05^a,b^
Monocytes (×10^3^/mL)	0.19 ± 0.02	0.32 ± 0.04	1.65 ± 0.29^a,b^	1.50 ± 0.20^a,b^

(a) represents significant difference between groups when compared to CG. (b) represents significant difference between groups when compared to DG. (d) represents significant difference between groups when compared to DMVG. Data are expressed as mean ± standard error of the mean (*n* = 10) and were analyzed by one-way ANOVA followed by Tukey's posttest (*p* < 0.05). CG: control group; DG: diet group; MVG: mechanical ventilation group; DMVG: diet mechanical ventilation group.

**Table 2:** Effects of diet and mechanical ventilation on cell recruitment to BALF.

	CG	DG	MVG	DMVG
Leukocytes (×10^3^/mL)	83.33 ± 4.41	119.00 ± 5.04^a^	204.4 ± 6.47^a,b^	228.0 ± 9.31^a,b,c^
Macrophages (×10^3^/mL)	78.46 ± 5.30	108.40 ± 4.74^a^	178.30 ± 7.03^a,b^	199.97 ± 8.80^a,b^
Neutrophils (×10^3^/mL)	1.61 ± 0.35	1.92 ± 0.37	8.00 ± 1.52^a,b^	8.13 ± 1.10^a,b^
Lymphocytes (×10^3^/mL)	3.26 ± 0.52	8.68 ± 1.01	18.10 ± 2.30^a,b^	19.90 ± 2.45^a,b^

(a) represents significant difference between groups when compared to CG. (b) represents significant difference between groups when compared to DG. (c) represents significant difference between groups when compared to MVG. Data are expressed as mean ± standard error of the mean (*n* = 10) and were analyzed by one-way ANOVA followed by Tukey's posttest (*p* < 0.05). CG: control group; DG: diet group; MVG: mechanical ventilation group; DMVG: diet mechanical ventilation group.

**Table 3:** Biomarkers of inflammation on pulmonary parenchyma.

	CG	DG	MVG	DMVG
CCL2 (pg/mL)	536.6 ± 95.15	446.7 ± 108.2	1896 ± 273.9^a,b^	1465 ± 237.6^a,b^
CCL5(pg/mL)	334.4 ± 56.37	176.4 ± 30.36	258.8 ± 69.23	316.5 ± 39.74
IL-17 (pg/mL)	1065 ± 33.61	1060 ± 31.40	790.9 ± 26.78^a,b^	884.2 ± 52.74^a,b^
IL-22 (pg/mL)	185.8 ± 38.4	207.6 ± 50.18	753 ± 96.97^a,b^	619.9 ± 95.65^a,b^
IL-10 (pg/mL)	3543 ± 150.70	3367 ± 99.34	2436 ± 33.12^a,b^	2820 ± 67.87^a,b^

(a) represents significant difference between groups when compared to CG. (b) represents significant difference between groups when compared to DG. Data are expressed as mean ± standard error of the mean (*n* = 10) and were analyzed by one-way ANOVA followed by Tukey's posttest (*p* < 0.05). CG: control group; DG: diet group; MVG: mechanical ventilation group; DMVG: diet mechanical ventilation group; IL-10: interleukin-10; IL-17: interleukin-17; IL-22: interleukin-22.

**Table 4:** Biomarkers of oxidative stress on pulmonary parenchyma.

	CG	DG	MVG	DMVG
SOD (U/mg ptn)	23.33 ± 1.98	28.83 ± 3.60	43.33 ± 7.18^a^	37.32 ± 2.19^a^
CAT (U/mg ptn)	1.01 ± 0.06	0.57 ± 0.07^a^	0.19 ± 0.02^a,b^	0.13 ± 0.01^a,b^
GSH/GSSG ratio	6.38 ± 1.64	5.47 ± 0.60	1.97 ± 0.18^a^	5.05 ± 1.52
TBARS (nmol/mg ptn)	2.42 ± 0.27	2.16 ± 0.21	5.85 ± 1.11^a,b,d^	2.77 ± 0.32
Protein carbonyl (nmol/mg ptn)	4.74 ± 0.89	3.45 ± 0.37	14.20 ± 1.64^a,b^	18.46 ± 4.34^a,b^

(a) represents significant difference between groups when compared to CG. (b) represents significant difference between groups when compared to DG. (d) represents significant difference between groups when compared to DMVG. Data are expressed as mean ± standard error of the mean (*n* = 10) and were analyzed by one-way ANOVA followed by Tukey's posttest (*p* < 0.05). CG: control group; DG: diet group; MVG: mechanical ventilation group; DMVG: diet mechanical ventilation group; CAT: catalase; GSH: glutathione reduced; GSSG: glutathione oxidized; SOD: superoxide dismutase; TBARS: thiobarbituric acid reactive substances.
